# Muscle Reactive Oxygen Species (ROS) Contribute to Post-Incisional Guarding via the TRPA1 Receptor

**DOI:** 10.1371/journal.pone.0170410

**Published:** 2017-01-19

**Authors:** Daisuke Sugiyama, Sinyoung Kang, Timothy J. Brennan

**Affiliations:** 1 Department of Anesthesia, Roy J. and Lucille A. Carver College of Medicine, University of Iowa, Iowa City, Iowa, United States of America; 2 Department of Pharmacology, Roy J. and Lucille A. Carver College of Medicine, University of Iowa, Iowa City, Iowa, United States of America; Hebrew University Hadassah Medical School, ISRAEL

## Abstract

**Background:**

Deep tissues and their afferents have unique responses to various stimuli and respond to injury distinctively. However, the types of receptors and endogenous ligands that have a key role in pain after deep tissue incision are unknown. TRPA1 has been shown to mediate pain-related responses in inflammation- and nerve injury-induced pain models. We hypothesized that TRPA1 has an important role in pain behaviors after deep tissue incision.

**Methods:**

The effect of various doses of intraperitoneal (i.p.) TRPA1 antagonist, HC-030031, on pain behaviors after skin + deep tissue incision of the rat hind paw was measured. *In vivo* reactive oxygen species (ROS)-imaging and hydrogen peroxide (H_2_O_2_) levels after incision were also evaluated. Separate groups of rats were examined for H_2_O_2_-evoked pain-related behaviors after injections into the deep tissue or the subcutaneous tissue.

**Results:**

Guarding pain behavior after skin + deep tissue incision was decreased by i.p. HC-030031. However, HC-030031 did not affect mechanical or heat responses after incision. Treatment either before or after incision was effective against incision-induced guarding behavior. ROS increased after skin + deep tissue incision in both the incised muscle and the skin. Tissue H_2_O_2_ also increased in both skin and muscle after incision. H_2_O_2_ injection produced pain behaviors when injected into muscle but not after subcutaneous injection.

**Conclusions:**

This study demonstrates that TRPA1 antagonist HC-030031 reduced spontaneous guarding pain behavior after skin + deep tissue incision. These data indicate that TRPA1 receptors on nociceptors are active in incised fascia and muscle but this is not evident in incised skin. Even though endogenous TRPA1 agonists like ROS and H_2_O_2_ were increased in both incised skin and muscle, those in skin do not contribute to nociceptive behaviors. This study suggests that endogenous TRPA1 ligands and the TRPA1 receptor are important targets for acute pain from deep tissue injury.

## Introduction

Postoperative pain continues to be a significant problem following surgery. To better understand the mechanisms for pain caused by surgery, we previously have generated a rat model of postoperative pain [1]. Using this model, we demonstrated that incision in skin + deep tissue caused greater guarding behavior and more spontaneous activity in nociceptors and dorsal horn neurons, compared to skin incision alone [2–4]. Various tissues and the afferents innervating these tissues have unique responses to injuries. Both preclinical and clinical studies suggest that deep tissue injury has an important role in postoperative pain [2, 5, 6]; however, the types of receptors and endogenous ligands that have a key role in incisional pain from deep tissues are currently unknown.

The Transient Receptor Potential Ankyrin 1 (TRPA1) channel is a member of the TRP channel family, and it has been shown to mediate pain-related responses in inflammation- and nerve injury-induced pain models [7]. TRPA1 is expressed in a subset of nociceptors expressing Transient Receptor Potential Vanilloid 1 (TRPV1) [7]. TRPA1 can be activated by noxious cold temperature, naturally occurring exogenous compounds, such as allyl isothiocyanate, cinnamaldehyde, and allicin and reactive oxygen species (ROS). [8–12]. ROS levels serve important signaling roles, including an adaptive response to stressful conditions. ROS are by-products of aerobic metabolism, and the most common ROS include superoxide anions (·O_2_^-^), hydroxyl radical and hydrogen peroxide (H_2_O_2_) [13]. ROS have an important role in wound healing and may contribute to postsurgical pain via the TRPA1 receptor.

Previously, we have shown that TRPV1-expressing nociceptors generate spontaneous guarding pain behavior after skin + deep tissue incision [14, 15]. However, pharmacological blockade or genetic knockout of TRPV1 did not suppress the guarding behavior [16, 17]. Therefore, TRPV1-containing nociceptors but not necessarily TRPV1 receptors play a major role in guarding behavior. We hypothesized that TRPA1 activation in wounds by ROS including H_2_O_2_ could contribute to pain behavior after incision. We examined the effect of the TRPA1 antagonist, HC-030031, on pain behaviors after skin + deep tissue incision. We also examined *in vivo* ROS-imaging and measured the levels of H_2_O_2_, an endogenous TRPA1 receptor ligand, in skin and deep muscle after incision.

## Materials and Methods

Procedures in this study were approved by The University of Iowa Animal Care and Committee (Approval number: 5011267), Iowa City, Iowa, USA and conformed to the NIH guide for the Care and Use of Laboratory Animals. Adult male Sprague-Dawley rats (200–320 g, Harlan, Indianapolis, IN) were housed in groups of 2 in clear plastic cages (40 x 60 x 30 cm) on fresh bedding with free access to food and water. The environment was controlled with a 12 hour light-dark cycle and a room temperature of 22.0 ± 2.0°C. The physical conditions of the animals were carefully monitored every weekday during the experiments. The clinical signs of illness included sustained weight loss, self-destructive behavior, abnormal reaction of the central nervous system, and any obvious functional injury. The animals did not show any signs of stress (except pain-related behavior) or illness throughout the experiment. One hundred and four rats were assigned for pain behavior after incision, 54 rats were assigned for *in vivo* ROS imaging, 18 rats were assigned for the H_2_O_2_ assay, and 37 rats were assigned for nociceptive behavior after H_2_O_2_ injection. Altogether, 213 rats were used in this study.

### Surgical incisions

This study used two types of incisions: (1) skin + deep tissue incision of the hind paw, which involves incision of the skin, underlying fascia, and the plantar flexor digitorum brevis muscle and (2) skin + deep tissue incision at the gastrocnemius muscle, which incised skin, underlying fascia, and divided one head of the gastrocnemius muscle. Detailed methods for performing these incisions were described in previous studies [1, 2, 18] Briefly, anesthesia was induced by placing the animal in a sealed plastic box containing 5% isoflurane mixed with air. During surgery, anesthesia was maintained with 1.5–2% isoflurane delivered through a nose cone. The skin of the surgical site was prepared with 10% povidone-iodine immediately before incision.

For skin + deep tissue incision of the hind paw, a 1-cm longitudinal incision was made and the underlying fascia and the plantar flexor digitorum brevis muscle were incised with a #11 surgical blade. Blunt curved forceps were then inserted through the incision into the muscle to further divide and retract the muscle. The muscle origin and insertion remained intact. The wound was then closed with two subcutaneous mattress sutures with 6–0 nylon on a P-1 needle (Ethicon, Somerville, NJ, USA).

For the skin + deep tissue incision at the gastrocnemius muscle, beginning 1 cm from the edge of the heel, a 2-cm longitudinal incision was made through the skin, underlying fascia, and the gastrocnemius muscle with a # 11 surgical blade. Grasping forceps were then inserted through the incision into one head of the gastrocnemius muscle to divide and retract the muscle. The muscle origin and insertion remained intact. The wound was then closed with three subcutaneous mattress sutures with 6–0 nylon. For the skin incision group, beginning 1 cm from the edge of the heel, a 2-cm longitudinal incision was made only through the skin overlying gastrocnemius muscle. The incised skin was then closed with three subcutaneous mattress sutures of 6–0 nylon.

### Incision-induced pain behaviors

Detailed methods for these behavioral tests were described previously [1, 2, 18]. Briefly, rats were first acclimated to the testing environment for 3 days. Then a baseline test was performed 1 day before incision. After hind paw incision, pain behaviors were measured up to 2 days after incision. The person performing the behavioral test was blinded to drug injected.

For guarding behavior, rats were placed individually on a small plastic mesh floor (grid 8 × 8 mm) covered with a clear plastic cage top (21 × 27 × 15 cm). Both incised and non-incised hind paws were closely observed during a 1-minute scoring period, and a score of 0, 1, or 2 was given. Zero was scored when the incised area (or corresponding area in the non-incised hind paw) was touching the mesh, and the area was blanched or distorted by the mesh; 1 was scored when the incised area touched the mesh without blanching or distortion; 2 for the position when the incised area was completely off of the mesh. We scored once every 5 minutes for 1 hour after incision. Therefore, a cumulative score was obtained by adding the 12 scores during the 1-hour testing period (0–24) for each hind paw. The guarding score was then obtained by subtracting the score of the incised hind paw from that of the non-incised hind paw.

For mechanical withdrawal threshold, rats were placed on a plastic mesh floor with 12 × 12 mm openings. Calibrated Semmes-Weinstein monofilaments (Stoelting, Wood Dale, IL, USA) were used for mechanical testing. The filaments were carefully applied from underneath the mesh to an area adjacent to the incision or a corresponding area in the non-incised hind paw. Starting with 13 mN, each filament was applied once until a withdrawal response was provoked. If the force of 228 mN was reached and there was still no withdrawal response, then 673 mN, the bending force of the next filament, was recorded as the withdrawal threshold. This test was performed for three times with at least a 5-minute interval between tests. The lowest force that elicited a response from the three tests was defined as the mechanical withdrawal threshold.

For heat withdrawal latency, rats were placed individually on a glass floor covered with a clear plastic cage. The experimental room temperature was maintained at 22 ± 2°C. Radiant heat from a 50-W projector lamp was applied to the incised hind paw from underneath the glass floor. The latency to evoke withdrawal was determined with a cutoff value of 20 seconds. Each rat was tested three times with an interval of at least 10 minutes. The average of the three trials was recorded as the heat withdrawal latency.

#### Experimental protocols for incision-induced pain behaviors

We evaluated the effect of various doses of intraperitoneal (i.p.) HC-030031 (75, 150 and 300 mg/kg) on the pain-related behaviors at several time points after skin + deep tissue incision of the hind paw. The control group received i.p. vehicle instead of HC-030031.

First, the effect of HC-030031, injected after hind paw incision, on guarding behavior was evaluated. Pre-incision baseline guarding was measured 1 day before incision. Plantar incision was made and guarding was measured 2 hours after incision. Then the animals received an i.p. injection of HC-030031 or vehicle. Guarding behavior was evaluated at multiple time points following drug administration up to postoperative day (POD) 2.

Then, the effect of HC-030031 on mechanical and heat hyperalgesia after skin + deep tissue incision of the hind paw was evaluated in one separate group of rats. After baseline responses were measured, incision was made and post-incision mechanical and heat responses were recorded. Then HC-030031 or vehicle was administered. Mechanical withdrawal threshold and heat withdrawal latency were measured at multiple time points through POD 2. The effect of HC-030031 on heat and mechanical responses was not further studied.

Two more sets of experiments were performed to further examine the effect of HC-030031 on guarding behavior after hind paw incision. First, we examined the effect of HC-030031, injected both on POD 0 and POD 1, on guarding behavior. After the baseline measurement, incision was made and guarding was measured 2 hours later. Then HC-030031 (vehicle, 75, 150 or 300 mg/kg) was injected and guarding measured twice. On the next day, pre-drug guarding was measured and drug administration was repeated followed by two more measurements.

Next, we evaluated the effect of HC-030031, injected immediately before the incision. Because there were little differences among doses in the second protocol, 300 mg/kg dose was not administered in this protocol. After the baseline measurement, rats were anesthetized, and HC-030031 (vehicle, 75 or 150 mg/kg) was injected immediately prior to hind paw incision. Guarding was measured several times on the day of incision through POD 2.

### *In vivo* reactive oxygen species imaging

In order to image skin and deep tissue for ROS-induced luminescence, we used gastrocnemius incision. The greater thickness of the leg region allowed us to study a superficial image that included skin and a deeper image that included muscle. This was not possible with the hind paw incision. Rats were assigned to three separate groups: (1) the gastrocnemius muscle incision group had a skin + deep tissue incision on their left leg, and the contralateral leg underwent sham incision and served as a control; (2) one group underwent gastrocnemius incision, and catalase was injected (1,000–2,500 IU) locally into the gastrocnemius incision before imaging; and (3) the skin-only incision group had the skin incision only on the left leg.

L-012-mediated *in vivo* ROS imaging was performed on POD 0, POD 1, and POD 7 in separate groups of rats for each day, thus, rats were imaged only once. During *in vivo* imaging, the rats were immobilized with anesthesia using isoflurane (1.5–2.5%) and injected with L-012 (25 mg/kg) administered subcutaneously. The imaging system (IVIS 200, Xenogen, CA, USA) consisted of a light-tight chamber equipped with a cooled CCD camera. The luminescent images were captured 5 minutes after L-012 injection. Catalase was injected locally 2 minutes before L-012 injection. Data acquisition was accomplished with Living Image^®^ software (Xenogen, CA, USA). Image exposure times were 1 minute. Light emission from the region of interest was quantified as photons/second · cm^2^ in a single plane (steradian, sr).

### Hydrogen peroxide assay

The H_2_O_2_ levels in gastrocnemius muscle and skin overlying the gastrocnemius muscle in incised and non-incised tissue were determined using a commercial fluorescence Amplex^®^ Red Hydrogen Peroxide assay kit (Invitrogen, OR, USA) [19]. Briefly, rats were studied on POD 0, POD 1 and POD 7 after incision of the gastrocnemius muscle or skin overlying the gastrocnemius muscle. Under deep anesthesia with isoflurane, the incised gastrocnemius muscle and skin were removed. Contralateral, non-incised muscle and skin were also removed as controls. The samples were rapidly frozen on an aluminum plate, which had been chilled in dry ice. After freezing the tissue, the samples were trimmed and homogenized in 50 mM phosphate buffer (pH 7.4) containing 5 mM sodium azide at 4°C for 60 seconds. Before the homogenate was centrifuged, a portion was saved for protein measurement. The homogenate was centrifuged at 4000 rpm at 4°C for 15 minutes. The supernatant obtained was frozen at -80°C until assayed for H_2_O_2_ levels. Samples and H_2_O_2_ standards were assayed spectrophotometrically at 560 nm using EnVision Multilabel Plate Reader (Perkin Elmer, MA, USA). The H_2_O_2_ levels were calculated using a standard curve and were normalized to tissue protein as determined by the DC Protein Assay Kit (Bio-Rad, CA, USA).

### Nociceptive behavior after injection of hydrogen peroxide

In order to evaluate H_2_O_2_-evoked pain-related behaviors in skin and deep tissue, we used injections into the gastrocnemius muscle or the subcutaneous tissue overlying gastrocnemius muscle. Rats were acclimated individually on a small plastic mesh floor covered with a clear plastic cage top for 1 hour per day for at least 2 days before testing. On the testing day, injection of H_2_O_2_ (100 mM, 0.6 ml) into the left gastrocnemius muscle or subcutaneous tissue overlying the gastrocnemius muscle was made using a 1-ml syringe with a 30-gauge needle. We observed that volumes of at least 0.8 ml of Evan’s Blue dye remained in the gastrocnemius muscle of injections. The control group was injected with intramuscular synthetic interstitial fluid (SIF, 0.6 ml) instead of H_2_O_2_. Rats were returned to the testing cage and observed for 1 hour. H_2_O_2_-induced nociceptive behavior in rats was recorded as total time spent flinching, lifting and licking of the hind leg.

The effect of locally-injected HC-030031 on H_2_O_2_-induced nociceptive behavior was also evaluated. Sequential injections of HC-030031 (50 mM, 0.3 ml) or vehicle (0.3 ml), followed by H_2_O_2_ (100 mM, 0.3 ml), were made into the gastrocnemius muscle. Therefore, the total injection volume was 0.6 ml, and the final concentration of H_2_O_2_ was 50mM. Immediately after injection, nociceptive behavior was recorded for 1 hour as described above.

The person performing the behavioral test was blinded to drug and dose. The person preparing drugs assigned these in an informal, random manner.

### Drugs

HC-030031, supplied by Hydra Biosciences (Cambridge, MA, USA), was suspended in 0.5% methylcellulose and administered i.p. in a volume of 10 ml/kg. L-012 was purchased from Tocris Bioscience (Avonmouth, Bristol, UK) and dissolved in ultra pure H_2_O for subcutaneous injection. Catalase from bovine liver (2,000–5,000 IU/mg protein) supplied as a range of activity, was purchased from Sigma-Aldrich (St Louis, MO, USA) and dissolved in phosphate-buffered saline (PBS; Gibco, Grand Island, NY, USA) for local injection at 1 mg/ml and pH 7.0. For local injections of HC-030031, drug was dissolved in 10% dimethylsulfoxide (DMSO; Molecular probes, Eugene, OR, USA) in PBS. H_2_O_2_ was purchased from Sigma-Aldrich (St. Louis, MO, USA), and diluted in SIF or PBS.

### Statistics

For continuous data, the Kolmogorov-Smirnov test of normality was used to determine whether the data values had normal distributions. Two-way ANOVA with repeated measures on one factor followed by Bonferroni’s post hoc test was used to analyze the guarding pain behavior, withdrawal latency to heat and the H_2_O_2_ detection assay. Non-parametric Friedman’s test followed by Kruskal-Wallis test with Dunn post hoc test was used to analyze the withdrawal threshold to mechanical stimulation. Two-way ANOVA with Bonferroni’s post hoc test was used to analyze the ROS-imaging. Unpaired t-test and one-way ANOVA with Bonferroni’s post hoc test were used to analyze the nociceptive behavior after injection of H_2_O_2_. Values of P < 0.05 were considered significant. Data were presented as mean ± standard error of the mean (SEM) or as median with range. All tests were conducted using GraphPad Prism (version 5.04, Graphpad Software, Inc., CA, USA).

## Results

### Incision-induced pain behaviors

**Fig 1:**
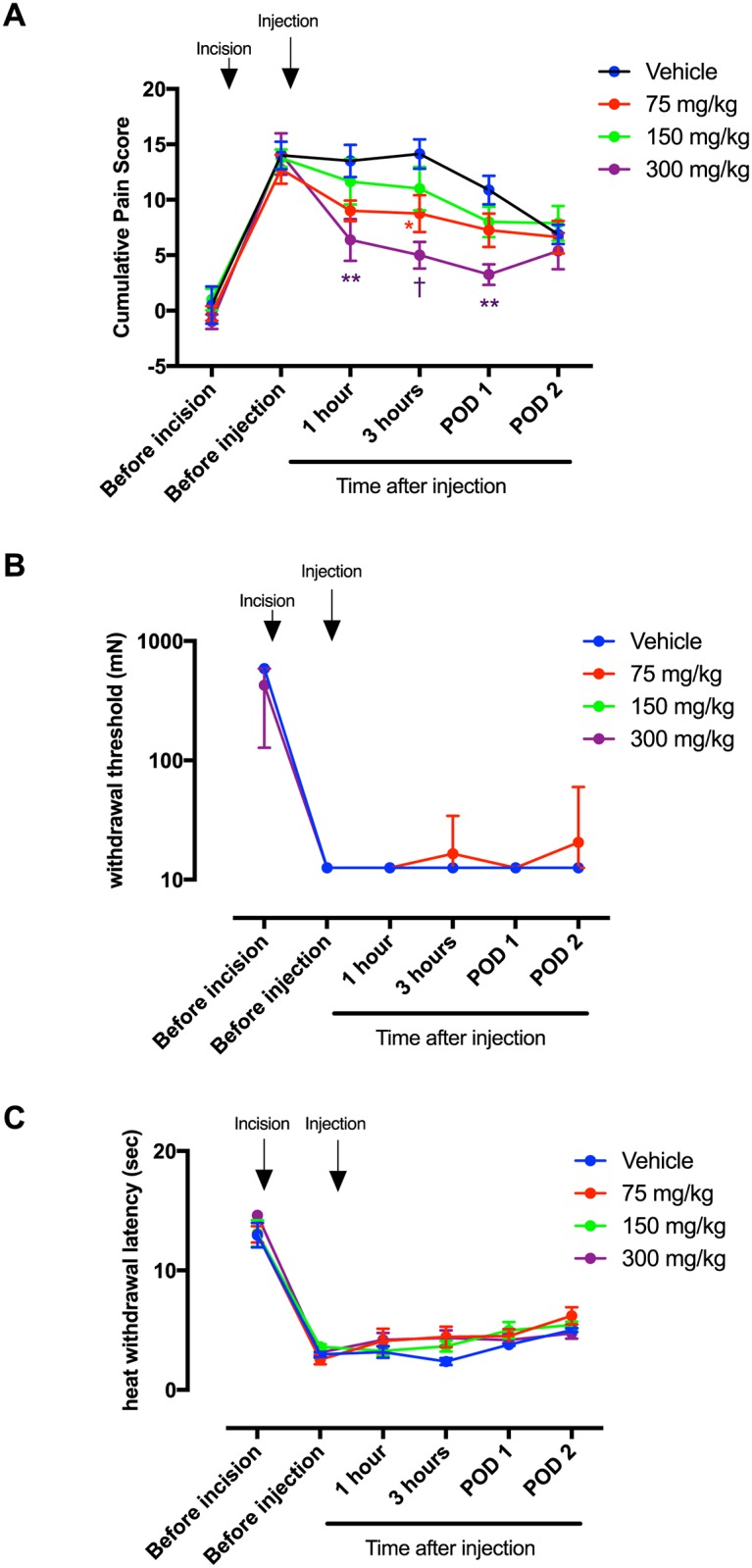
Effect of intraperitoneal (i.p.) administration of HC-030031 on pain behaviors of rats after skin + deep tissue incision. (A) Guarding pain behavior. The results are presented as mean and standard error of the mean (SEM) for eight rats in each group. Two-way ANOVA with repeated measures on one factor (interaction factor: F_24, 210_ = 1.64, P = 0.0360) followed by Bonferroni’s post hoc test for comparing the mean cumulative pain score at each time point among groups. (B) Withdrawal threshold to punctate stimuli applied to the hind paw. The results are presented as median with range for six rats in each group. Non-parametric Friedman’s test (Fr = 17.02, P = 0.0019) followed by Kruskal-Wallis test with Dunn post hoc test for between-group comparisons at each time point. (C) Withdrawal latency to heat stimulation. The results are presented as mean and SEM. Two-way ANOVA with repeated measures on one factor (interaction factor: F_24, 150_ = 1.163, P = 0.2855) followed by Bonferroni’s post hoc test for comparing the mean withdrawal latency at each time point among groups. * P < 0.05, ** P < 0.01, † P < 0.001 compared with the vehicle group at each time point. POD = postoperative day.

Skin + deep tissue incision of the hind paw induced guarding pain behavior one hour after incision in all groups of rats prior to the injection of HC-030031 or vehicle (Fig 1A). Compared with the vehicle group, rats treated with 75 mg/kg of HC-030031 showed less guarding pain behavior at 3 hours after drug injection (8.8 ± 1.7, P = 0.0192). Rats administered with 300 mg/kg of HC-030031 showed less guarding pain behavior at 1 hour (6.4 ± 1.9, P = 0.0007),s (5.0 ± 1.2, P < 0.0001) after drug injection and again on POD 1 (3.3 ± 0.9, P = 0.0003), compared to the vehicle group (Fig 1A).

We tested these same doses in separate groups of rats for heat and mechanical responses after incision. Skin + deep tissue incision induced mechanical hyperalgesia in all groups (Fig 1B). Compared to the vehicle group, no difference in withdrawal threshold was present in all three dose groups of rats treated with HC-03003, at any time after drug administration.

For heat responses, skin + deep tissue incision reduced the heat withdrawal latency in all groups (Fig 1C). No differences in heat withdrawal latency were evident in the three groups treated with HC-030031 compared to vehicle at any time after drug administration.

**Fig 2:**
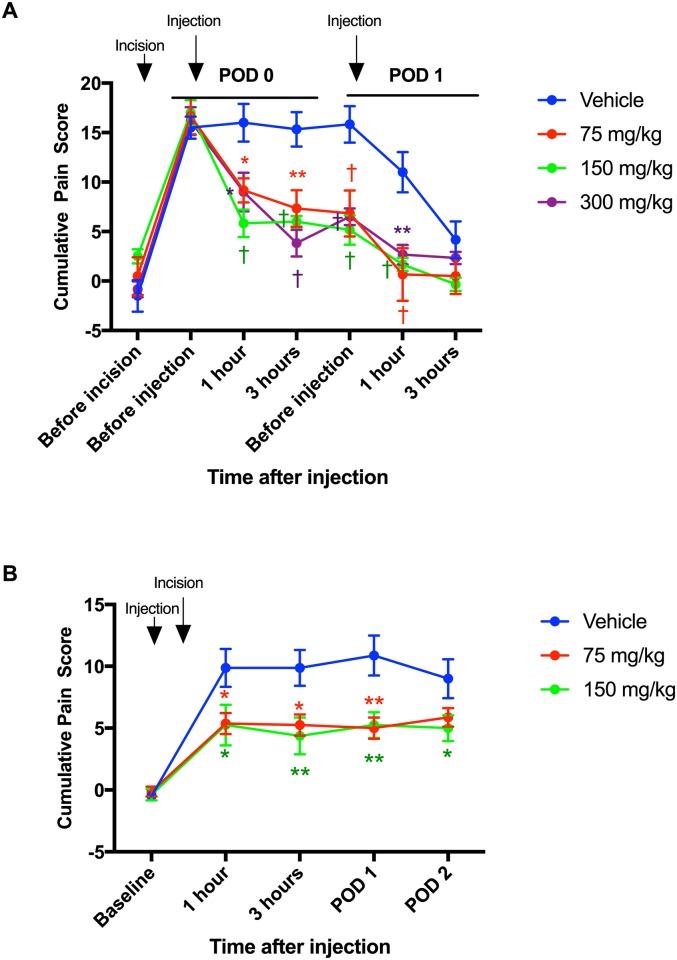
Additional studies on effect of intraperitoneal (i.p.) administration of HC-030031 on guarding pain. (A) HC-030031 was injected two times, once on postoperative day (POD) 0 and POD 1. The results are presented as mean and SEM for six rats in each group. Two-way ANOVA with repeated measures on one factor (interaction factor: F_24, 160_ = 3.026, P < 0.0001) followed by Bonferroni’s post hoc test for comparing the mean cumulative pain score at each time point among groups. (B) HC-030031 was injected immediately prior to plantar incision. The results are presented as mean and SEM for eight rats in each group. Two-way ANOVA with repeated measures on one factor (interaction factor: F_10, 105_ = 2.95, P = 0.0026) followed by Bonferroni’s post hoc test for comparing the mean cumulative pain score at each time point among groups. * P < 0.05, ** P < 0.01, † P < 0.001 compared with vehicle-injected rats at each time point.

We treated rats twice with HC-030031, after incision and on POD 1, and tested only against guarding behaviors. As shown in Fig 2A, 75 mg/kg of HC-030031 attenuated guarding behavior at 1 hour (9.2 ± 1.2, P = 0.0130) and 3 hours (7.3 ± 1.8, P = 0.0022) after the first injection, before the second injection on POD 1 (6.8 ± 2.3, P = 0.0004) and 1 hour after second injection (0.7 ± 2.7, P < 0.0001). The 150 mg/kg group had less guarding behavior at 1 hour (5.8 ± 1.4, P < 0.0001) and 3 hours (6.0 ± 0.6, P = 0.0002) after the first injection, before injection on POD 1 (5.2 ± 1.5, P < 0.0001) and 1 hour after second injection (1.7 ± 0.7, P = 0.0002). The 300 mg/kg group exhibited less guarding behavior at 1 hour (9.0 ± 1.9, P = 0.0102) and 3 hours (3.8 ± 1.4, P < 0.0001) after the first injection, before injection on POD 1 (6.5 ± 0.8, P = 0.0002) and 1 hour after the second injection (1.7 ± 0.7, P = 0.0002) (Fig 2A).

We also administered HC-030031 against guarding once immediately prior to the skin + deep tissue incision of the hind paw. Because drug effect was similar among doses in Fig 2A, only 75 and 150 mg/kg were administered. Compared with the vehicle group, rats treated with 75 mg/kg of HC-030031 had less guarding behavior at 1 hour (5.4 ± 0.9, P = 0.0199), and 3 hours after incision (5.3 ± 0.9, P = 0.0159) and also on POD 1 (5.0 ± 0.9, P = 0.0014). Rats administered 150 mg/kg group showed less guarding behavior at 1 hour (5.3 ± 1.7, P = 0.0159), 3 hours (4.4 ± 1.5, P = 0.0030), POD 1 (5.3 ± 1.0, P = 0.0023) and POD 2 (5.0 ± 1.0, P = 0.0463) (Fig 2B).

### *In vivo* reactive oxygen species imaging

**Fig 3:**
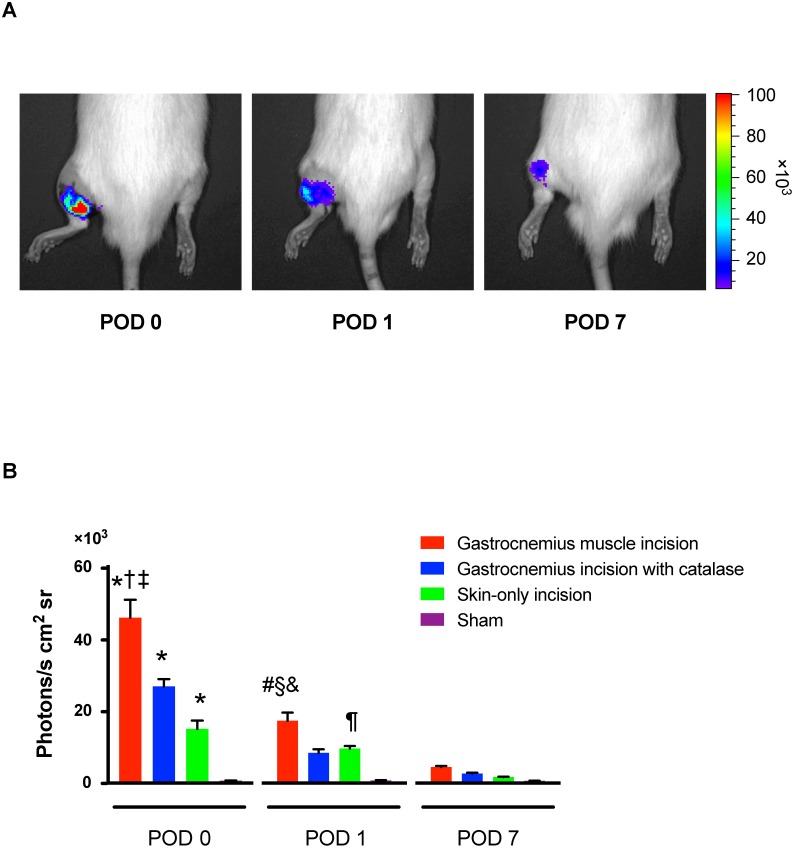
*In vivo* reactive oxygen species (ROS)-imaging with L-012 after incision in rats. (A) Examples of *in vivo* imaging after gastrocnemius muscle incision. (B) Average luminescence intensity in *in vivo* ROS-imaging on gastrocnemius muscle incision, gastrocnemius incision with catalase (1,000–2,500 IU), skin-only incision, and sham. The results are presented as mean and SEM for 6 rats in each group. * P < 0.0001 compared with sham, † P < 0.0001 compared with gastrocnemius incision with catalase, ‡ P < 0.0001 compared with skin-only incision, # P < 0.0001 compared with sham, § P = 0.0159 compared with gastrocnemius incision with catalase, & P = 0.0490 compared with skin-only incision, ¶ P = 0.0166 compared with sham. Two-way ANOVA (interaction factor: F_6, 30_ = 22.56, P < 0.0001) followed by Bonferroni's post hoc tests.

A prominent luminescent signal could be detected at the incised leg in both a superficial scan of skin and a deeper scan of muscle. Almost no signal was detected from contralateral, sham-operated, non-incised tissue (Fig 3A). Compared to the sham group, chemiluminescence intensity from the gastrocnemius muscle incision was increased to 46,000 ± 4,400 photons/s · cm^2^ · sr on POD 0 (P < 0.0001), and 17,000 ± 2,200 photons/s · cm^2^ · sr on POD 1 (P < 0.0001) (Fig 3B). The luminescent probe signal from the skin-only incision group was increased to 15,000 ± 2,300 photons/s · cm^2^ · sr on POD 0 (; P < 0.0001) and 10,000 ± 700 photons/s · cm^2^ · sr on POD 1 (P = 0.0166), compared with sham group (Fig 3B). Scanning deeper layers that included muscle produced a greater signal than scanning superficially at skin on POD 0 and POD 1. Injection of catalase into the incised muscle before deep tissue imaging reduced the luminescent probe signal from the gastrocnemius incision on POD 0 and POD 1; the signal was 27,000 ± 2,000 photons/s · cm^2^ · sr (P < 0.0001) on POD 0, and 8,000 ± 1,000 photons/s · cm^2^ · sr (P = 0.0159) on POD 1 (Fig 3B). In summary, both the skin and muscle incision increased the luminescent probe signal on POD 0 and POD 1 compared to sham and these signal were not different from sham on POD 7 (Fig 3B).

### Hydrogen peroxide assay

**Fig 4:**
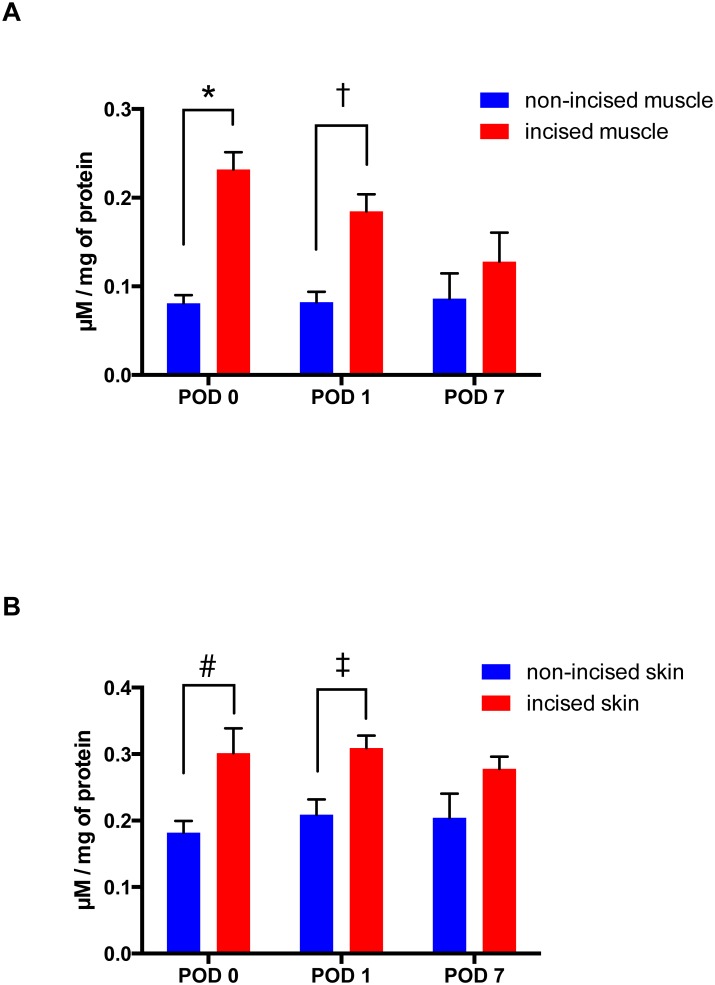
Effects of the incision of skin and muscle on tissue hydrogen peroxide (H_2_O_2_) levels in rats. (A) H_2_O_2_ content after incision in gastrocnemius muscle using the Amplex^®^ Red Hydrogen Peroxide assay kit. The results are presented as mean and SEM for 6 rats in each group. Two-way ANOVA with repeated measures on one factor (interaction factor: F_2, 15_ = 2.328, P = 0.1317, Time factor: F_2, 15_ = 3.890, P = 0.0436, Group factor: F_1, 15_ = 22.58, P = 0.0003) followed by Bonferroni’s post hoc test. * P < 0.0001 compared with non-incised muscle on POD 0, † P = 0.0074 compared with non-incised muscle on POD 1. (B) H_2_O_2_ content after incision of skin overlying the gastrocnemius muscle. The results are presented as mean and SEM for 6 rats in each group. Two-way ANOVA with repeated measures on one factor (interaction factor: F_2, 15_ = 0.5907, P = 0.5663, Time factor: F_2, 15_ = 0.2134, P = 0.8103, Group factor: F_1, 15_ = 31.71, P < 0.0001) followed by Bonferroni’s post hoc test. # P = 0.0105 compared with incised skin on POD 0, ‡ P = 0.0373 compared with incised skin on POD 1.

Spectrophotometric analysis of the H_2_O_2_ levels in gastrocnemius muscle and skin homogenates revealed a significantly higher content of the H_2_O_2_ in the incised tissues (Fig 4). The H_2_O_2_ levels in incised gastrocnemius muscle was 284% greater on POD 0 (P < 0.0001), 226% greater on POD 1 (P = 0.0074), and 151% greater on POD 7 (P = 0.5612), compared to non-incised muscle (Fig 4A). The H_2_O_2_ levels after incision in skin overlying the gastrocnemius muscle were 165% greater on POD 0 (P = 0.0105), 148% greater on POD 1 (P = 0.0373), and 136% greater on POD 7 (P = 0.1819), compared to non-incised skin (Fig 4B).

### Nociceptive behavior after injection of hydrogen peroxide

**Fig 5:**
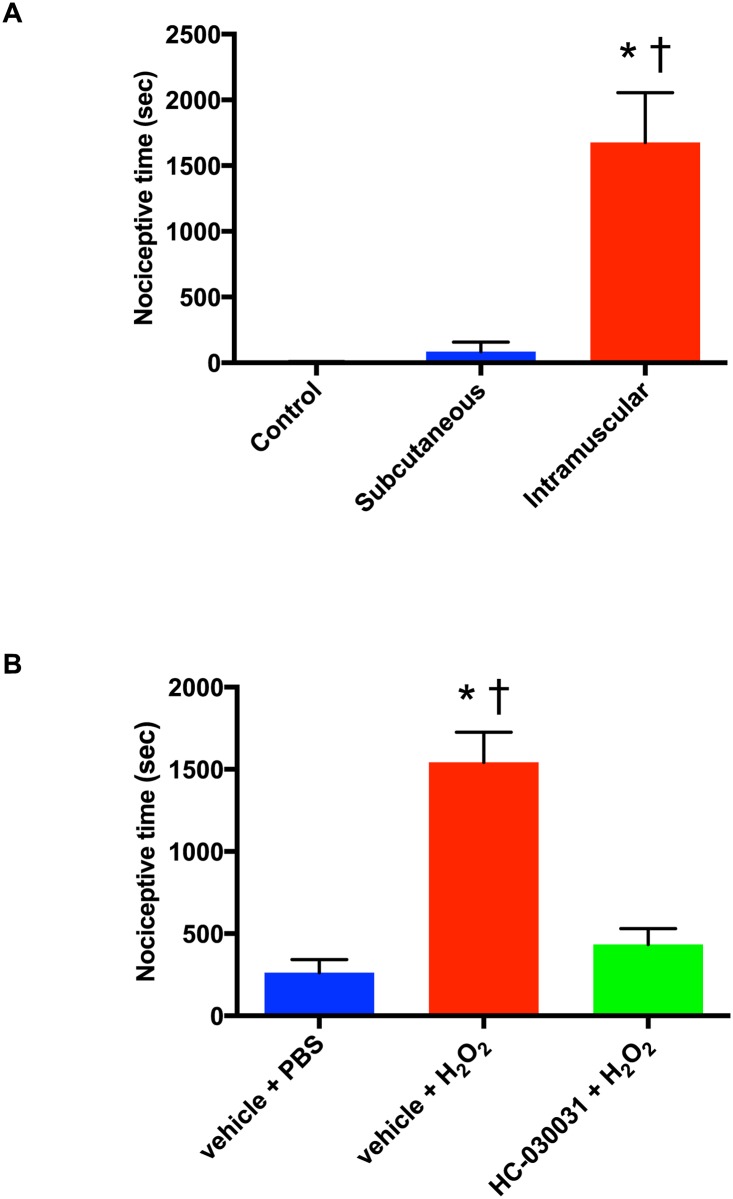
H_2_O_2_-induced nociceptive behavior in rats as total time spent flinching, lifting and licking of the hind leg. (A) Spontaneous nociceptive behavior after intramuscular (n = 6) or subcutaneous (n = 5) injection of H_2_O_2_ (100 mM, 0.6 ml), or intramuscular injection of synthetic interstitial fluid (0.6 ml) (n = 6). * P = 0.0004 compared with control group, † P = 0.0010 compared with subcutaneous H_2_O_2_ injection group by one-way ANOVA followed by post-hoc Bonferroni’s test. (B) Effects of local pre-injection of a TRPA1 antagonist (HC-030031; 50 mM, 0.3 ml, n = 6) on nociceptive behavior caused by intramuscular injection of H_2_O_2_ (100 mM, 0.3 ml, n = 7) vs. vehicle group (n = 7). * P < 0.0001 compared with vehicle + PBS injection group, † P < 0.0001 compared with HC-030031 + H_2_O_2_ injection group by one-way ANOVA followed by post-hoc Bonferroni’s test. All data are expressed as means ± SEM.

Intramuscular injection of H_2_O_2_ (100 mM, 0.6 ml) produced greater nociceptive behavior (1,675 ± 378 sec) compared to control (7 ± 3 sec; P = 0.0004) and subcutaneous H_2_O_2_ injection (83 ± 74 sec; P = 0.0010) (Fig 5A). Pre-treatment with locally injected TRPA1 antagonist HC-030031 (50 mM, 0.3 ml) significantly reduced nociceptive behavior induced by H_2_O_2_ (100 mM, 0.3 ml) (P < 0.0001 vs. vehicle + H_2_O_2_ group) (Fig 5B).

## Discussion

In the present study, we have demonstrated that guarding pain behavior after skin + deep tissue incision was decreased by parenterally administered HC-030031, a TRPA1 antagonist. However, HC-030031 did not affect mechanical or heat responses after incision. Treatment either before or after surgery was effective against guarding. In some cases, the effect to reduce guarding persisted after a single dose into POD 1. A second dose of HC-030031 on POD 1 also reduced guarding. We showed that ROS increased after skin + deep tissue incision in both the gastrocnemius muscle and the skin. The increase in ROS in the gastrocnemius muscle after incision was reduced by local injection of catalase, suggesting that the increase in ROS was in part attributable to an increase in H_2_O_2_. Furthermore, we demonstrated that tissue H_2_O_2_ increased in both skin and muscle after incision. However, H_2_O_2_ injection produced pain behaviors when injected into muscle but not after subcutaneous injection.

### 1. Effect on guarding, not on heat and mechanical hyperalgesia

We have demonstrated that guarding pain behavior, but not mechanical and heat hyperalgesia, was decreased by the TRPA1 antagonist HC-030031 (Fig 1). We have previously characterized guarding pain behavior after plantar incision and shown that guarding was evident only when incision of deep muscle tissue was included [2, 3]. Skin incision alone was insufficient to produce significant guarding behavior. In addition, deep muscle and fascia injury produced spontaneous activity in nociceptors and nociceptive dorsal horn neurons, but this was much less apparent after skin incision [2, 3]. Finally, we suggested that guarding and spontaneous activity in nociceptive pathways are a correlate to the pain at rest in postoperative patients [2, 3]. HC-030031 did not affect heat and mechanical responses for which skin incision is sufficient for the full, early hypersensitivity after incision [20, 21].

In previous studies, guarding was inhibited by local anesthetic infiltration, administration of clinically relevant doses (0.03–0.1 mg/kg) of parenteral morphine, nerve growth factor (NGF) sequestration and capsaicin-induced nociceptor desensitization (administered by infiltration or proximal perineural application) [15–17, 22–24].

### 2. ROS, H_2_O_2_ and wounds

H_2_O_2_ is a major ROS and is a relatively stable ROS that can diffuse into tissues and cross cell membranes [25]. H_2_O_2_ is an early signal of incisional tissue injury and functions as an oxidant with antibacterial and cytotoxic effects [26]. H_2_O_2_ has been shown to be generated by skin incision but has not been studied in injured deep tissues. ROS including H_2_O_2_ have several roles in skin including recruiting leukocytes and supporting wound healing [27, 28]. Recent studies have shown that TRPA1 acts as a molecular detector of cellular stress, including ROS [29, 30].

This study indicates that H_2_O_2_ and likely other ROS, previously identified in cutaneous wounds and shown to contribute to wound healing, are factors producing deep tissue pain after incisions. Intramuscular Injection of H_2_O_2_ causes nociceptive pain but no such behaviors were evident after subcutaneous injection (Fig 5). Studies by others using another TRPA1 agonist, formalin, have not specified cutaneous versus deep tissue differences in nociception after injection [31, 32]. Intraplantar injection of H_2_O_2_ caused brief nociceptive pain [19], but specific subcutaneous versus deep tissue injections were not studied and would be difficult in such small spaces like the hind paw. In other previous studies, after intraplantar injection of H_2_O_2_, nociceptive behavior was short-lasting (< 5 min), which is consistent with our finding after subcutaneous injection over the gastrocnemius [30, 33, 34]. In humans, cutaneous wounds are frequently flushed or rinsed with much higher concentrations of H_2_O_2_ (880 mM concentration of commercially-available 3% H_2_O_2_).

Malin et al. demonstrated that functional expression of TRPA1 using TRPA1 agonist-induced Ca^2+^ transients was significantly greater in cultured dorsal root ganglia neurons innervating muscle compared to those innervating skin [35]. In addition, TRPA1 responses in dorsal root ganglia neurons were potentiated by growth factors but changes in responsiveness varied depending upon the organ. and Dorsal root ganglia neurons innervating muscle were more likely to exhibit NGF-induced potentiation of TRPA1 responses compared to dorsal root ganglia neurons innervating skin.

### 3. The role of TRPA1 in other incisional pain studies

Our study indicates that TRPA1 has a substantial role on guarding behavior after incision of deep tissue not subcutaneous tissue. This is because TRPA1 probably has different roles in various organs. In previous studies, pharmacological blockade of TRPA1 reversed mechanical hypersensitivity and on-going pain-related behavior in different models of inflammatory and neuropathic pain [17, 36, 37].

Barabas et al. studied the contribution of TRPA1 to mechanical hypersensitivity following skin incision of the hind paw in TRPA1 knockout and wild-type mice [38]. In their study, skin incision produced mechanical hypersensitivity in TRPA1-knockout mice to similar levels measured in wild-type mice. Thus, they concluded that TRPA1 did not mediate mechanical hypersensitivity following cutaneous surgical incision in mice. Our data are in agreement.

Wei et al. examined local and spinal TRPA1 after skin + deep tissue incision of the hind paw in the rat [39]. In their study, i.p. or ipsilateral intraplantar treatment with TRPA1 antagonists reduced guarding and mechanical hypersensitivity after skin + deep tissue incision. Intrathecal drug treatment attenuated mechanical responses but not guarding. They concluded that TRPA1 contributed to guarding behavior and mechanical hyperalgesia after incision. We did not observe an effect of HC-030031 on mechanical responses. However, the increased ROS after deep tissue incision in our study (Fig 3), and the nociceptive effect of H_2_O_2_ after injection into muscle but not skin, explains and connects the contribution of TRPA1 to behaviors dependent on incised deep tissue. This is in part agreement with Wei et al. [39].

## Conclusion

This study demonstrates that TRPA1 antagonist HC-030031 reduced spontaneous guarding pain behavior after skin + deep tissue incision, but did not reduce mechanical and heat responses. These data indicate that TRPA1 receptors are contributing to incisional pain in incised fascia and muscle but not in incised skin. Endogenous TRPA1 agonists like ROS and H_2_O_2_ were increased in both incised skin and muscle, however, injection of H_2_O_2_ into muscle caused nociceptive behaviors but not injection into skin. Together, this study suggests that endogenous TRPA1 ligands and the TRPA1 receptor are important targets for acute pain from deep tissue injury.
